# Evolving Trends in Pediatric Inflammatory Bowel Disease Management in Japan: A Decade of Nationwide Data

**DOI:** 10.1002/jgh3.70175

**Published:** 2025-05-14

**Authors:** Miki Urushiyama, Kunio Tarasawa, Rintaro Moroi, Hideya Iwaki, Yusuke Hoshi, Hiroshi Nagai, Yusuke Shimoyama, Takeo Naito, Fumihiko Kakuta, Hisashi Shiga, Shin Hamada, Yoichi Kakuta, Kiyohide Fushimi, Yoshitaka Kinouchi, Daiki Abukawa, Kenji Fujimori, Atsushi Masamune

**Affiliations:** ^1^ Division of Gastroenterology Tohoku University Hospital Sendai Japan; ^2^ Department of Health Administration and Policy Tohoku University Graduate School of Medicine Sendai Japan; ^3^ Department of Gastroenterology and Hepatology Miyagi Children's Hospital Sendai Japan; ^4^ Department of Health Policy and Informatics Tokyo Medical and Dental University Graduate School of Medicine and Dental Sciences Bunkyo Japan

**Keywords:** big data, biologics, inflammatory bowel disease, pediatric patients

## Abstract

**Aims:**

This study aimed to investigate the trends in pediatric inflammatory bowel diseases (IBD) management in Japan over the past decade.

**Methods:**

We retrospectively analyzed data from Japan's nationwide database from 2012 to 2022. Patients aged ≤ 15 years diagnosed with Crohn's disease (CD) or ulcerative colitis (UC) were included. Trends in the use of biologics, capsule endoscopy, total parenteral nutrition (TPN), elemental diets, surgery, and granulocyte and monocyte apheresis (GMA) were examined using the Cochrane–Armitage and Jonckheere–Terpstra trend tests.

**Results:**

Among the 8037 and 6153 pediatric UC and CD admissions, respectively, the use of biologics increased significantly (CD: from 46.0% to 53.6%; UC: from 15.0% to 33.0%, *p* < 0.0001). The use of capsule endoscopy in pediatric patients with CD increased markedly from 6.6% to 16.7% (*p* < 0.0001), whereas TPN use decreased from 8.4% to 3.0% (*p* < 0.0001). Surgery rates for patients with CD remained at approximately 5%, whereas those for patients with UC decreased (from 3.7% to 1.7%, *p* = 0.002). Elemental diets for pediatric patients with CD increased (from 54.4% to 66.2%, *p* < 0.0001). The use of GMA decreased significantly in patients with UC (from 12.1% to 2.7%, *p* < 0.0001).

**Conclusion:**

The use of biologics and capsule endoscopy has increased in pediatric patients with IBD, whereas the use of more invasive treatments has decreased. These trends suggest a shift toward less invasive and more targeted therapeutic strategies in managing pediatric patients with IBD in Japan.

## 1 Introduction

Inflammatory bowel disease (IBD), which comprises Crohn's disease (CD) and ulcerative colitis (UC), is a chronic relapsing condition that results in substantial morbidity. The incidence and prevalence of adult‐onset IBD are increasing globally [1]. In addition to adult‐onset IBD, the incidence of pediatric‐onset IBD has been increasing worldwide, with Non‐Caucasian populations, including Asian populations, recently showing an exponential increase in the incidence of pediatric‐onset IBD [2, 3].

In Japan, substantial changes in diagnostic and therapeutic strategies for children with IBD have been observed in clinical practice. In addition to conventional therapies (5‐aminosalicylic acid, elemental diet, and steroids), biologics, including infliximab and adalimumab, have recently been approved and have been administered to both pediatric and adult patients [4]. Furthermore, capsule endoscopy has been approved for clinical use in pediatric patients with IBD. This minimally invasive technique is effective for diagnosing IBD and has also become a primary method for monitoring IBD because of its low physical burden and ease of use [5].

While previous studies have examined IBD trends in adult patients [6, 7], nationwide analyses focusing specifically on pediatric patients with IBD are limited. To address this gap, our study utilized a large‐scale national database to evaluate recent trends in pediatric IBD diagnosis and treatment. Given the increasing global incidence of pediatric IBD, understanding Japan's clinical trends may provide valuable insights for other countries, particularly Asian countries, where similar epidemiological shifts are occurring.

This study aimed to elucidate the clinical practices and trends in the treatment and diagnosis of pediatric patients with IBD using a nationwide database, by comparing these practices and trends with those observed in adult patients with IBD.

## 2 Methods

### 2.1 Diagnosis Procedure Combination (DPC) System

The DPC database, introduced in 2003, is a medical claims database for acute care hospital admissions in Japan. The system was adopted by 1764 hospitals in 2022, encompassing 85% of acute care beds (approximately 480 000 beds) in Japan [8]. The DPC database contains information regarding patient demographics, diagnoses, comorbidities upon admission, medications, surgeries, and procedures. Disease diagnosis was categorized as “main diagnosis,” “main disease triggering admission,” and “most resource‐consuming diagnosis.” Physicians entered these patient diagnoses into the database according to the International Classification of Diseases, Tenth Revision (ICD‐10) codes. Several studies have reported the diagnostic validity of the DPC database [9, 10, 11].

### 2.2 Eligible Admissions and Extraction of Data

We collected the administrative claims data of inpatients discharged from more than 1000 participating hospitals. This study included patients with UC or CD who were admitted to a DPC‐participating hospital between April 2012 and March 2022. Eligible patients were identified using a DPC diagnosis containing the phrases “ulcerative colitis” or “Crohn's disease” in their main diagnosis, main disease triggering admission, or the most resource‐consuming diagnosis. ICD10 codes for UC and CD are “K51” and “K50,” respectively. We excluded entries for suspected cases of diverticulitis by filtering results containing the word “suspicious.”

### 2.3 Data Collection

We collected data on the patients, their clinical characteristics, and the procedures they underwent from the DPC database. These data included variables such as age, sex, hospital type (academic vs. non‐academic), urgency of hospitalization (emergency or elective), length of hospital stay, associated medical costs, specific medications used for managing IBD (systemic steroids or molecular targeted drugs), surgery for IBD, granulocyte and monocyte apheresis (GMA) for UC, central venous catheter placement for total parenteral nutrition (TPN) for IBD, and capsule endoscopy for CD. The molecular targeted drugs included the following: infliximab, adalimumab, golimumab, vedolizumab, ustekinumab, and tacrolimus. In addition, we evaluated the biosimilars of infliximab and adalimumab. Surgeries, including colectomies, small bowel resections, seton drain insertion, strictureplasties, and stoma creations (colostomies and ileostomies), were also identified using the K‐code in the database.

GMA is an extracorporeal circulation therapy that selectively absorbs and removes activated neutrophils, monocytes, and macrophages. GMA is typically conducted for up to 10 sessions as an induction therapy and has demonstrated high efficacy [12, 13]. GMA has also been approved as a maintenance therapy for UC in Japan since 2021. Although infliximab and adalimumab are currently approved for pediatric patients with IBD in Japan, golimumab, vedolizumab, ustekinumab, and tacrolimus are not.

### 2.4 Data Analyses

In Japan, the healthcare system is structured in such a way that individuals ≤ 15 years of age (junior school students) are typically referred to pediatric departments, whereas those > 15 years (high school students) are referred to adult internal medicine departments. A survey conducted in Japan reported that approximately two‐thirds of adult gastroenterologists considered 16 years to be the ideal age for patient transfer [14]. Based on these considerations, we adopted 15 years as the cut‐off age for pediatric patients in this study.

**FIGURE 1:**
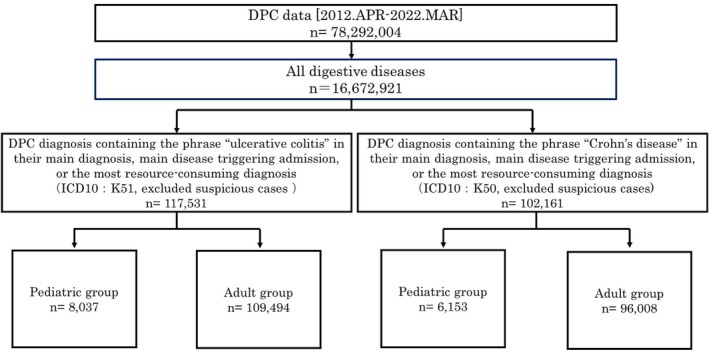
Flowchart of patient data extraction.

We divided eligible admissions based on age into two groups: ≤ 15 years (pediatric group) and > 15 years (adult group), and subsequently classified each group into patients with UC or CD (Figure 1). In the CD analysis, the number of patients who underwent capsule endoscopies, received biologics, received elemental diets, underwent surgery, and received TPN was calculated every 2 years and divided by the number of admissions in the same age group to calculate the penetration rate. In the UC analysis, the number of patients who received prednisolone, tacrolimus, and biologics and who underwent surgery and GMA was extracted, and the penetration rate of each item was calculated. We subsequently investigated trends in the actual number and penetration rate of each item over the past 10 years. The Cochrane–Armitage trend test was used to evaluate temporal changes in medicine prescription patterns, diagnostic investigations, and admission rates in pediatric patients with IBD. Additionally, the Jonckheere–Terpstra trend test was used to assess trends in the length of hospital stay over time.

The threshold for statistical significance was set at *p* < 0.05. All analyses were performed using JMP Pro17 software (SAS Institute, Tokyo, Japan).

## 3 Results

### 3.1 Background of Study Population

We collected data on 8037 and 6153 eligible admissions of pediatric patients with UC and CD, respectively. The characteristics of the study population are summarized in Table 1. The academic hospital admission rate was significantly higher in the pediatric CD group than in the adult CD group (49.1% vs. 37.6%, *p* < 0.0001), whereas the emergency admission rate in the pediatric CD group was significantly lower than that in the adult CD group (23.4% vs. 32.1%, *p* < 0.0001). The same trend was observed in the pediatric UC group (academic admission: 46.4% vs. 25.8%, *p* < 0.0001; emergency admission: 43.6% vs. 50.9%, *p* < 0.0001). The mortality rate was marginally higher in the adult group than in the pediatric group (CD: 0.0005% vs. 0.003%, *p* < 0.0001; UC: 0.0006% vs. 0.008%, *p* < 0.0001, respectively).

**TABLE 1:** Clinical characteristics of IBD.

	Crohn's disease			Ulcerative colitis		
	Pediatric group, *n* = 6153	Adult group, *n* = 96 008	*p*	Pediatric group, *n* = 8037	Adult group, *n* = 109 494	*p*
Sex (male/female) *n* (%)	3955 (64.3)/2198 (35.7)	66 385 (69.1)/29 623 (30.9)	< 0.0001	4364 (54.3)/3673 (45.7)	62 552 (57.1)/46 942 (42.9)	< 0.0001
Age (mean ± SD) (years)	11.7 ± 3.54	39.9 ± 14.7	< 0.0001	11.8 ± 3.2	47.3 ± 19.4	< 0.0001
Academic admission, *n* (%)	3025 (49.1)	36 105 (37.6)	< 0.0001	3728 (46.4)	28 212 (25.8)	< 0.0001
Emergency admission, *n* (%)	1437 (23.4)	30 825 (32.1)	< 0.0001	3508 (43.6)	55 723 (50.9)	< 0.0001
Hospital stay (mean ± SD) (days)	14.4 ± 25.3	14.7 ± 25.0	< 0.0001	19.4 ± 33.7	21.1 ± 23.3	< 0.0001
Medical costs (mean ± SD) (yen)	790 375 ± 1 250 756	784 890 ± 935 993	< 0.0001	982 663 ± 1 761 278	1 016 198 ± 1 126 254	< 0.0001
Death, *n* (%)	3 (0.0005)	286 (0.003)	< 0.0001	5 (0.0006)	852 (0.008)	0.0002

### 3.2 Clinical Practice and Trends of Diagnosis and Treatment of CD

The comparative data on clinical practice differences between pediatric and adult patients with CD is summarized in Table 2.

**TABLE 2:** Comparative data on clinical practice differences between pediatric and adult patients with Crohn's disease.

		2012–2013	2014–2015	2016–2017	2018–2019	2020–2021	*p*
Number of admissions	Pediatric	914	1067	1305	1316	1551	
	Adult	19 510	20 632	20 317	18 803	16 746	
The rate of CD admissoins for all digestive diseases (%)	Pediatric	1.1	1.2	1.6	1.0	1.7	< 0.0001^a^
	Adult	0.6	0.6	0.6	0.6	0.5	< 0.0001^a^
TPN rate (%)	Pediatric	5.6	4.5	4.2	4.9	5.1	< 0.0001^a^
	Adult	20.2	22.4	23.6	25	26.7	< 0.0001^a^
Surgery rate (%)	Pediatric	8.4	7.1	5.6	4.3	3	0.95^a^
	Adult	13.2	12.7	11.3	8.7	8.2	< 0.0001^a^
Hospital stay (day)	Pediatric	16.1	16.2	14.5	12.9	13.4	0.0008^b^
	Adult	16.3	15.6	14.6	13.6	13.3	0.12^b^
CE rate (%)	Pediatric	6.6	9.6	12.8	16.1	16.8	< 0.0001^a^
	Adult	1.5	2.0	2.5	3.0	3.6	< 0.0001^a^
Biologics prescription rate (%)	Pediatric	46.0	47.3	50.0	51.9	53.6	< 0.0001^a^
	Adult	50.8	47.1	44.5	41.7	39.3	< 0.0001^a^
ED prescription rate (%)	Pediatric	54.5	62.6	71.3	63.3	66.2	< 0.0001^a^
	Adult	39.5	40.8	43.5	42.2	43.8	< 0.0001^a^

Abbreviations: CD, Crohn's disease; CE, capsule endoscopy; ED, elemental diet; TPN, total parental nutrition.

^a^ Cochrane–Armitage trend test.

^b^ Jonckheere–Terpstra trend test.

Among the 102 161 admissions, the number of admissions for pediatric CD increased marginally from 914 in 2012–2013 to 1551 in 2020–2021, whereas the number of admissions for adult CD decreased from 20 317 in 2016–2017 to 16 746 in 2020–2021 (Figure S1a).

**FIGURE 2:**
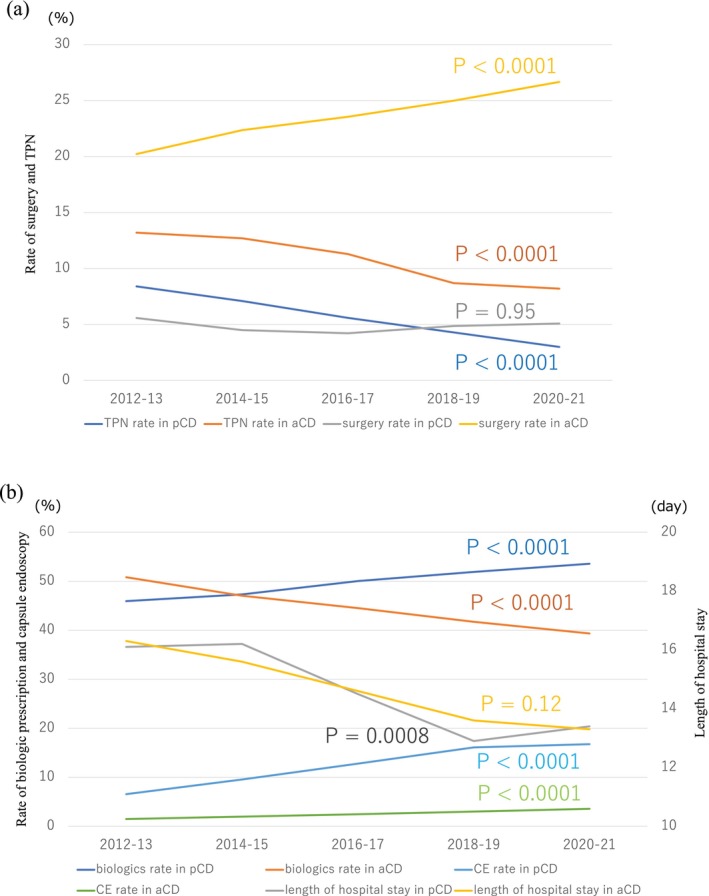
Changes in clinical practice, diagnostic investigations, and treatment for Crohn's disease over the previous decade. (a) The rate of TPN usage decreased in both pediatric and adult patients (*p* < 0.0001, *p* < 0.0001, respectively). The rate of surgery for CD admissions in pediatric patients remained constant at approximately 5% (Cochrane–Armitage trend test, *p* = 0.95). (b) The rate of biologics administration for pediatric patients with CD increased (*p* < 0.0001). The rate of capsule endoscopy in pediatric CD admissions has increased (*p* < 0.0001). The length of hospitalization in pediatric patients with CD significantly decreased (Jonckheere–Terpstra trend test, *p* = 0.0008). aCD: adult Crohn's disease; pCD: pediatric Crohn's disease.

The rate of CD admission as a proportion of admissions for all digestive diseases significantly increased in pediatric patients (*p* < 0.0001) but decreased in adult patients (*p* < 0.0001) (Figure S1a). The TPN usage rate decreased in both pediatric and adult patients (pediatric CD: from 8.4% in 2012–2013 to 3% in 2020–2021, *p* < 0.0001; adult CD: from 13.2% in 2012–2013 to 8.2% in 2020–2021, *p* < 0.0001) (Figure 2a). The rate of surgery for patients with CD during admission remained constant at approximately 5% for pediatric CD, whereas the rate for adult CD increased over the last decade—from approximately 20% in 2012–2013 to approximately 25% in 2020–2021(*p* < 0.0001) (Figure 2a). The length of hospital stay for pediatric patients with CD significantly decreased from 16.1 days in 2012–2013 to 13.4 days in 2020–2021 (*p* = 0.0008), whereas that of adult patients with CD did not significantly decrease (*p* = 0.12) (Figure 2b).

The rate of undergoing capsule endoscopy during admission among patients with CD increased from 6.6% in 2012–2013 to 16.7% in 2020–2021, particularly in pediatric patients (*p* < 0.0001) (Figure 2b). The rate of elemental diet prescriptions increased in both pediatric and adult patients (pediatric CD: from 54.4% in 2012–2013 to 66.2% in 2020–2021, *p* < 0.0001; adult CD: from 39.5% in 2012–2013 to 43.8% in 2020–2021, *p* < 0.0001) (Figure S1b). The rate of biologic administration in pediatric patients with CD increased over the last decade from 46.0% in 2012–2013 to 53.6% in 2020–2021 (*p* < 0.0001), whereas it decreased for adult patients with CD from 50.8% in 2012–2013 to 39.3% in 2020–2021 (*p* < 0.0001) (Figure 2b). The number of prescriptions for all biologics, particularly infliximab, increased in clinical practice for pediatric patients with CD (Figure S1c); moreover, the number of biosimilar prescriptions was marginally higher in pediatric patients with CD (Figure S1d).

The rate of capsule endoscopy usage increased significantly with advancing patient age (*p* = 0.0003) (Figure S3). The Cochrane–Armitage trend test indicated a significantly positive trend, with the rate of biologic administration increasing with pediatric patient age (Figure S4a) (*p* < 0.0001).

### 3.3 Clinical Practice and Trends of Diagnosis and Treatment of UC

The comparative data on the differences in clinical practice between pediatric and adult patients with UC is summarized in Table 3.

**TABLE 3:** Comparative data on clinical practice differences between pediatric and adult patients with ulcerative colitis.

		2012–2013	2014–2015	2016–2017	2018–2019	2020–2021	*p*
Number of admissions	Pediatric	1334	1437	1731	1666	1869	
	Adult	21 558	23 188	23 462	21 571	19 715	
The rate of UC admissoins for all digestive diseases (%)	Pediatric	1.6	1.7	2.1	1.2	2.1	0.025^a^
	Adult	0.7	0.7	0.7	0.7	0.6	< 0.0001^a^
Prednisolone prescription rate (%)	Pediatric	48.5	45.9	44.7	44.8	48.5	0.96^a^
	Adult	47.8	46.0	47.3	48.9	51.1	< 0.0001^a^
Tacrolimus prescription rate (%)	Pediatric	8.5	10.9	11.7	14.5	9.4	0.093^a^
	Adult	9.4	9.1	8.9	8.2	7.6	< 0.0001^a^
Biologics prescription rate (%)	Pediatric	15.1	15.6	21.5	26.8	33.4	< 0.0001^a^
	Adult	19.7	19.2	21.2	23.9	28.4	< 0.0001^a^
Surgery rate (%)	Pediatric	3.7	2.4	2.8	2.5	1.7	0.0020^a^
	Adult	7.9	7.5	7.7	7.5	6.9	0.0009^a^
Hospital stay (day)	Pediatric	22.2	20.1	20.2	18.9	16.6	0.0002^b^
	Adult	22.8	21.4	20.9	20.4	19.7	0.002^b^
GMA rate (%)	Pediatric	12.1	11.8	7.2	4.9	2.7	< 0.0001^a^
	Adult	17.8	16.0	15.2	12.3	9.8	< 0.0001^a^

Abbreviations: GMA, granulocyte and monocyte apheresis; UC, ulcerative colitis.

^a^ Cochrane–Armitage trend test.

^b^ Jonckheere–Terpstra trend test.

Among the 117 531 admissions, the number of pediatric UC admissions remained constant (Figure S2a). The rate of pediatric UC admissions as a proportion of admissions for all digestive diseases increased from 1.6% in 2012–2013 to 2.1% in 2020–2021 (*p* = 0.25) (Figure 2a).

**FIGURE 3:**
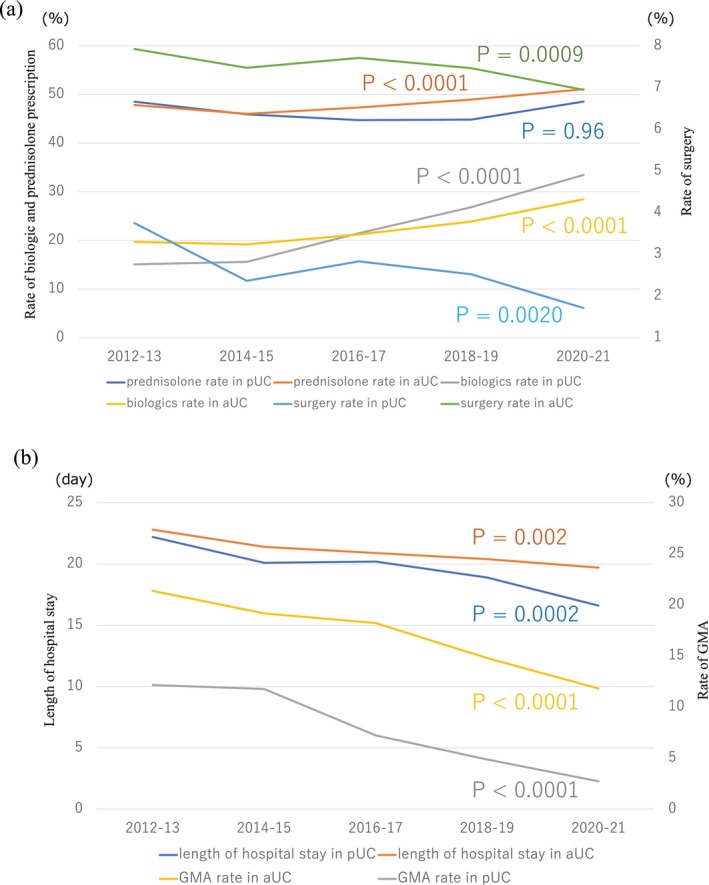
Clinical practice and transitions in the diagnosis and treatment of ulcerative colitis over the past decade. (a) The rate of prednisolone prescriptions for pediatric patients with UC remained constant at approximately 45% (*p* = 0.96). Rate of prescriptions for biologics increased (*p* < 0.0001). The rate of surgery decreased in pediatric patients (*p* = 0.0020). (b) Length of hospital stay for both pediatric and adult patients decreased (*p* = 0.0002, *p* = 0.002, respectively). The rate of GMA usage in UC admissions decreased significantly for both pediatric and adult patients (*p* < 0.0001 and *p* < 0.0001, respectively). aCD: adult Crohn's disease; pCD: pediatric Crohn's disease.

The rate of prednisolone prescriptions for pediatric patients with UC remained unchanged at approximately 45% (*p* = 0.96), whereas in adult patients, it increased from 47.8% in 2012–2013 to 51.1% in 2020–2021 (*p* < 0.0001) (Figure 3a). Although the rate of prescribing tacrolimus for pediatric patients increased marginally and peaked in 2018–2019, this difference was insignificant (*p* = 0.093) (Figure S2b).

The rate of prescribing biologics increased from 15% in 2012–2013 to 33% in 2020–2021, particularly among pediatric patients (*p* < 0.0001) (Figure 3a). The number of prescriptions for all biologics, particularly infliximab, increased in pediatric patients (Figure S2c), and a marginal increase in the number of prescriptions for biosimilars was observed (Figure S2d). The rate of surgery for both pediatric and adult patients decreased (pediatric UC: from 3.7% in 2012–2013 to 1.7% in 2020–2021, *p* = 0.0020; adult UC: from 7.9% in 2012–2013 to 6.9% in 2020–2021, *p* = 0.0009) (Figure 3a). The rate of biologic administration increased with increasing patient age (Cochrane–Armitage trend test, *p* < 0.0001) (Figure S4b).

The length of hospital stay for both pediatric and adult patients decreased (pediatric patients: from 22.2 days in 2012–2013 to 16.6 days in 2020–2021, *p* = 0.0002; adult patients: from 22.8 days in 2012–2013 to 19.7 days in 2020–2021, *p* = 0.002) (Figure 3b). The rate of GMA usage in UC admissions decreased significantly in both pediatric and adult patients (pediatric UC: 12.1% in 2012–2013 to 2.7% in 2020–2021, *p* < 0.0001; adult UC: 17.8% in 2012–2013 to 9.8% in 2020–2021, *p* < 0.0001) (Figure 3b).

## 4 Discussion

This study was a cross‐sectional analysis of the trends in hospitalization, treatment patterns, and surgical rates in pediatric patients with CD and UC over the previous decade. Its findings highlight temporal changes in management that reflect evolving clinical practices. To the best of our knowledge, this is the first study to statistically evaluate trends in the clinical management of patients with IBD, including treatment and diagnostic investigations of pediatric patients, using a Japanese nationwide database.

Our study revealed that less invasive diagnostic investigations and treatment modalities are increasingly being used in clinical practice in Japan for pediatric patients with CD. Although balloon‐assisted enteroscopy, magnetic resonance enterography, and enteroclysis are commonly used to investigate small bowel lesions in clinical practice, these procedures impose a high burden on pediatric patients [15]. In contrast, capsule endoscopy is less invasive, safer, and more tolerable in pediatric patients [16]. These advantages may have contributed to an increase in capsule endoscopy use among pediatric patients with CD. Another less invasive treatment modality for pediatric patients with CD is the use of an elemental diet. Low side effect rates are an advantage of the elemental diet [17, 18] and may have contributed to its high prescription rates. There are substantial differences in the frequency of elemental diet usage among different regions and practice methods [19, 20]. Although this study could not reveal the prescription volume and practice methods owing to the nature of the DPC database, pediatric patients' acceptance of an elemental diet in Japan appears to be favorable. Intestinal ultrasounds (IUSs) are conducted globally due to their usefulness in monitoring intestinal inflammation with low burdens. Through close monitoring, IUSs are expected to be useful in improving long‐term outcomes [21]. However, despite several studies reporting the usefulness of IUS [22, 23] in clinical practice in Japan, its use remains limited. Further investigations are warranted.

Our study also showed that interventions and treatments with a higher physical burden are decreasing. One example is the decreasing use of TPN in pediatric patients. Although TPN demonstrates a high probability of effectiveness, it requires a long hospitalization period. Furthermore, TPN can lead to potential catheter‐related complications [24, 25], which may decrease its usage rate. Furthermore, the use of biologics, described below, can contribute to the reduction in TPN because of their high efficacy. Consequently, the decrease in TPN use may have contributed to the reduced hospitalization times observed in this study.

Regarding the transition of treatment for pediatric patients with CD, another notable finding is that the prescription rate and the number of administrations of biologics, particularly infliximab, have increased over the past decade. This result indicates that biologics, including anti‐tumor necrosis factor α, play a crucial role in the treatment of pediatric and adult patients with CD in Japan [26]. Several new biologics, other than anti‐tumor necrosis factor α, have been added to pediatric IBD therapy in recent years, although they are not covered by insurance in Japan. These new agents affect the induction and remission rates [27, 28, 29]. The increased use of biologics may have contributed to the reduced hospitalization times and decreased use of TPN because of their high induction and maintenance efficacy, which ensures better nutritional conditions. Biologics are anticipated to lead to patient drug acceptance through treat‐to‐target strategies in both pediatric and adult patients [30].

The therapeutic options for UC have evolved over the last decade, with the most remarkable change being the increased use of biologics, although the prescription rate of prednisolone remains unchanged. This increase in the biologic administration rate might be attributable to the approval of biologics for pediatric patients and the high clinical need in pediatric patients with acute severe UC. Systemic steroid therapy remains the first‐line treatment, and the approval of biologics for pediatric patients may contribute to the increase in the biologic administration rate for UC. Furthermore, a review revealed that the incidence of severe acute attacks in childhood UC is considerably higher than that observed in adults (30–40% vs. 15%) [31]. These results indicate the high clinical need for biologics in pediatric patients with UC. However, our results also demonstrate that the surgery rate in pediatric UC significantly decreased. The short‐term surgery rate in acute severe UC declined from between 40% and 70% to approximately 10%–20% after the approval of infliximab and tacrolimus [32]. Previously, steroid‐refractory cases were primarily managed through surgery; however, with the advent of biologics, these patients can now achieve better disease control without resorting to surgical interventions. Biologics might reduce the need for surgery in pediatric patients with UC. The reduction in surgery rates may indirectly reflect this shift in treatment strategies, where biologics have become a key therapeutic option for managing severe UC, thereby decreasing the need for surgical interventions.

Our analysis demonstrates that the number of biosimilar prescriptions for pediatric patients with UC has increased and their use has become widespread. Another study using a nationwide database other than the DPC database reported similar results [6].

There are several possible explanations for the decline in GMA use. Although GMA is safe, well‐tolerated, and effective [33], some pediatric patients may experience difficulty in undergoing GMA as it requires catheter insertion and several weeks of hospitalization. Therefore, it is hypothesized that the increased use of biologics, which require shorter hospital stays than GMA administration, has resulted in fewer opportunities for its selection as a therapeutic option for pediatric patients with UC. Examinations and therapeutic options that burden patients with UC and CD may possibly be avoided in clinical practice.

Understanding the differences in clinical practices and treatment trends between pediatric and adult patients with IBD is crucial for adult gastroenterologists to ensure a seamless transition of care from the pediatric to the adult healthcare settings. This knowledge may contribute to optimizing long‐term disease management and improving patient outcomes. For example, our results revealed several differences in clinical practice, such as the biologic usage and surgery rates between pediatric and adult patients with CD. Additionally, the rising use of capsule endoscopy highlights the importance of promoting minimally invasive diagnostic approaches in pediatric patients. These trends suggest a need for continued updates in clinical guidelines and resource allocation to ensure optimal care for pediatric patients with IBD.

This study had several limitations. First, this study is a retrospective analysis using a claims database, and the possibility of selection bias cannot be ruled out due to the inherent limitations of such databases. Second, the DPC database does not contain details of patient conditions, such as the age at onset of IBD, the period when the condition (IBD) worsened, endoscopic and histopathological findings, laboratory data, computed tomography findings, or information on intestinal and extraintestinal manifestations. Consequently, we were unable to directly define “high‐risk” or “severe” cases based on clinical criteria. The DPC database also lacks information regarding genetic analyses and the age of onset of IBD, which prevented us from differentiating cases of monogenic IBD and conducting analysis focusing on early onset IBD and very early onset IBD. Third, the outpatient data, including the observation period and number of cases, were incomplete compared to the inpatient data because of the nature of the DPC database. Therefore, we excluded the outpatient data from this study. Fourth, DPC‐participating hospitals were typically acute care and relatively large‐volume hospitals. In addition, a report indicated that IBD treatment shifted to the outpatient setting following the introduction of new biologics [34]. Therefore, these data do not necessarily reflect all patients with IBD. Furthermore, if patients were transferred to another hospital, they could not be followed up.

## 5 Conclusion

The use of biologics demonstrated an increasing trend, whereas invasive treatments and examinations exhibited a tendency toward reduced application in pediatric patients with IBD. Understanding these trends is essential for improving long‐term disease management and ensuring a seamless transition of care from pediatric to adult healthcare settings. Our findings highlight the need for continued updates in clinical guidelines and resource allocation to optimize pediatric IBD management.

## Ethics Statement

The study protocol was reviewed and approved by the Ethics Committee of Tohoku University Graduate School of Medicine (approval number: 2022‐1‐412). The requirement for informed consent was waived owing to the use of anonymized patient data. The need for informed consent was waived by the Ethics Committee of Tohoku University Graduate School of Medicine (approval number: 2022‐1‐412).

## Conflicts of Interest

The authors declare no conflicts of interest.

## Supporting information


**Figure S1.** Changes in Crohn’s disease management. (a) Number and rate of admissions of patients with CD as a proportion of admissions for all digestive diseases. The rate of admission of patients with CD for all digestive diseases significantly increased in both pediatric and adult patients (*p* < 0.0001 and *p* < 0.0001, respectively). (b) The rate of elemental diet prescriptions increased for pediatric and adult patients with CD (*p* < 0.0001, *p* < 0.0001, respectively). (c) The number of prescriptions for all biologics, particularly infliximab, had increased. (d) The number of biosimilar prescriptions for pediatric patients increased marginally.
**Figure S2.** Changes in ulcerative colitis management. (a) The number of admissions for pediatric patients did not change. The admission rates of pediatric patients with UC as a proportion of admissions for all digestive diseases decreased (*p* < 0.0001). (b)The rate of tacrolimus prescriptions did not show an increasing tendency (*p* = 0.092). (c) The number of prescriptions for all biologics, particularly infliximab, has increased. (d) The number of biosimilar prescriptions for pediatric patients increased marginally.
**Figure S3.** Correlation between patient age at admission and the use of capsule endoscopy. The Cochran–Armitage trend test demonstrated that the rate of capsule endoscopy usage increased significantly with advancing patient age (*p* = 0.0003).
**Figure S4.** Trend of biologic administration in pediatric patients with IBD. (a) Patients with CD aged > 10 years exhibited a relatively stable and high rate of biologic administration over time. In contrast, the administration rates of biologics in patients aged ≤ 10 years showed a gradual increase over time. Furthermore, the Cochrane–Armitage trend test indicated a significant positive trend, with the rate of biologic administration increasing as patient age advanced (*p* < 0.0001). (b) Among pediatric patients with UC, the rate of biologic administration demonstrated an increasing trend with advancing patient age over time. This age‐dependent increase in biologic usage was further confirmed by the Cochrane–Armitage trend test, which revealed a statistically significant association (*p* < 0.0001).
