# Supplementary figures and images for: Evolving Trends in Pediatric Inflammatory Bowel Disease Management in Japan: A Decade of Nationwide Data
Source: JGH Open. 2025 May 14;9(5):e70175. doi: 10.1002/jgh3.70175 (PMC12078194; doi:10.1002/jgh3.70175)

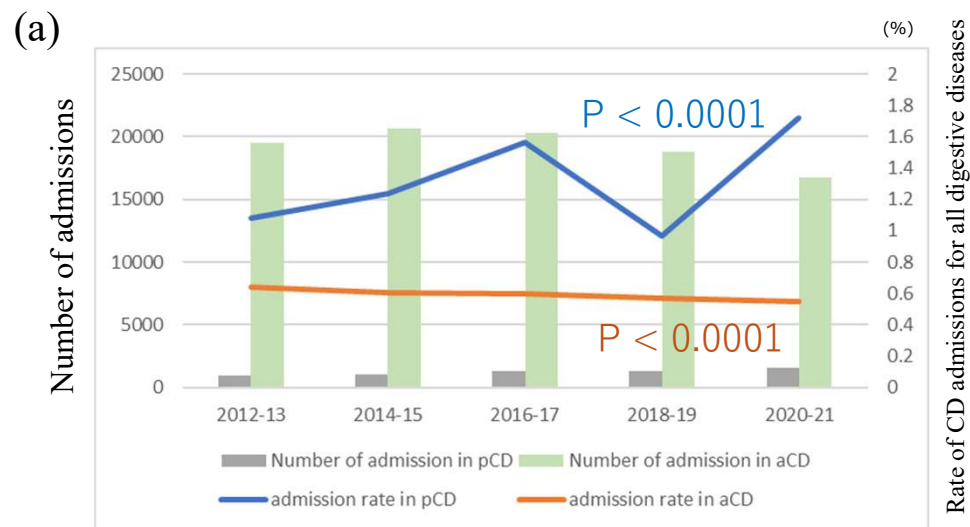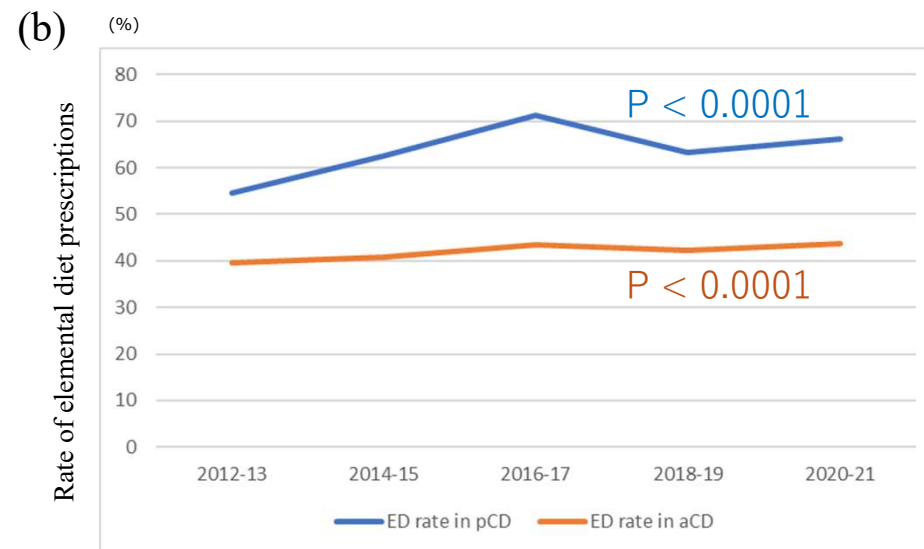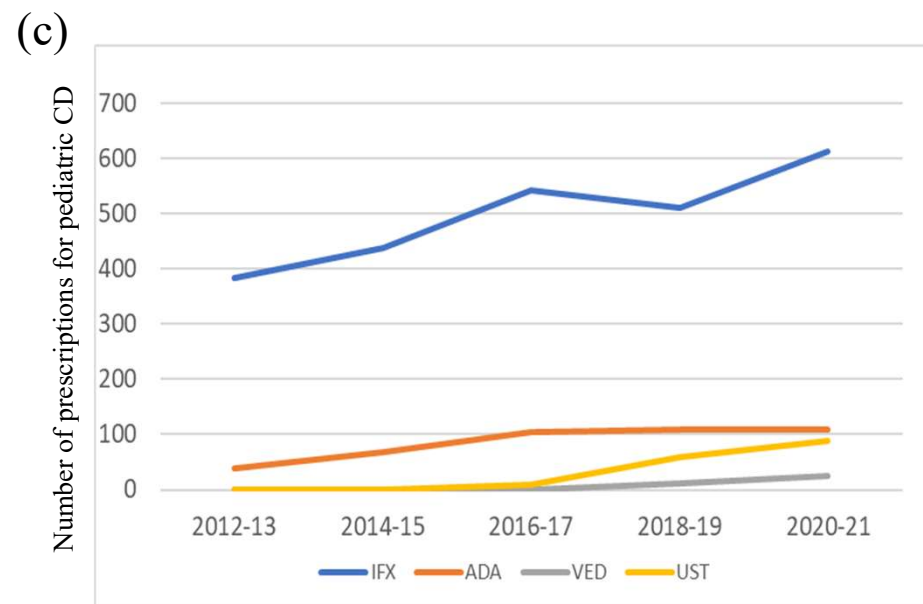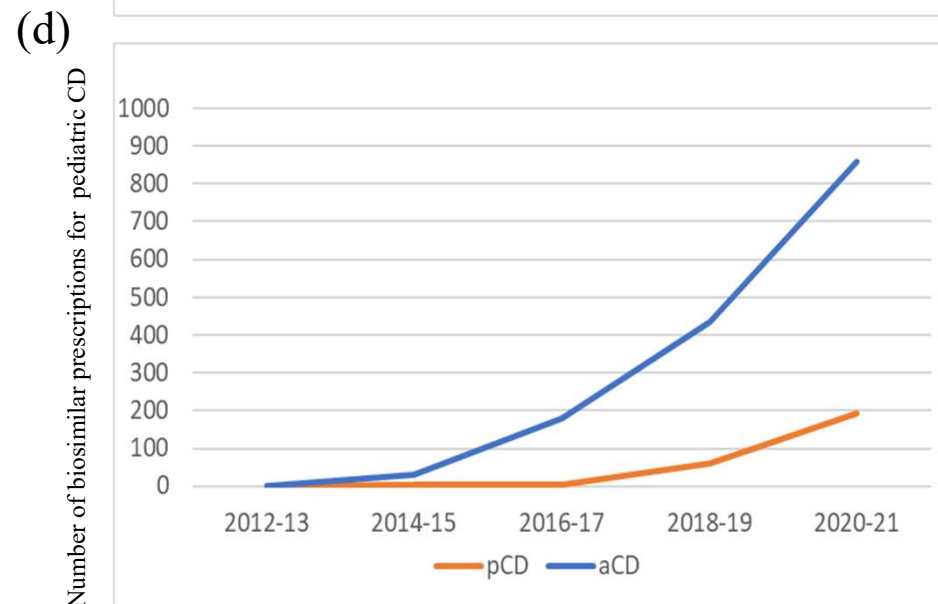

Supplementary figure 1

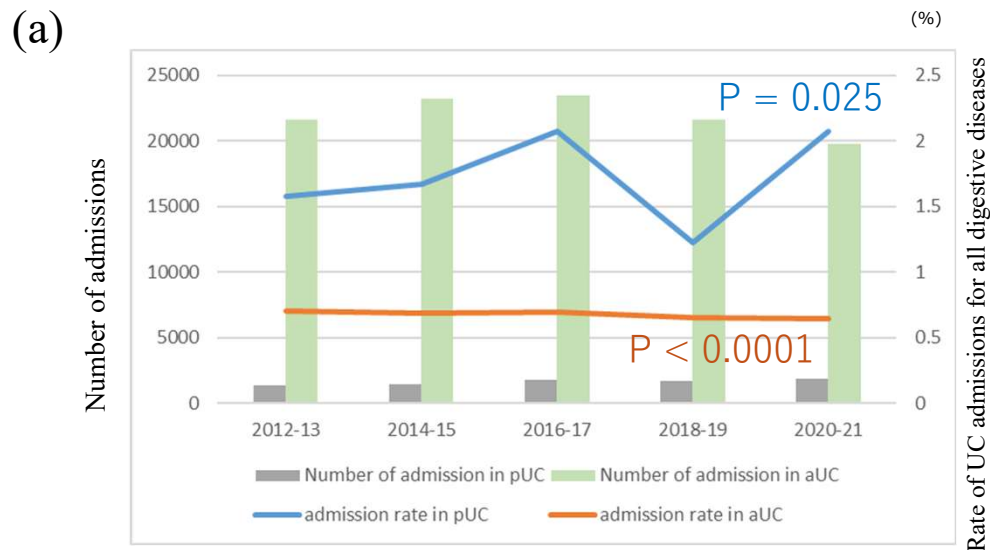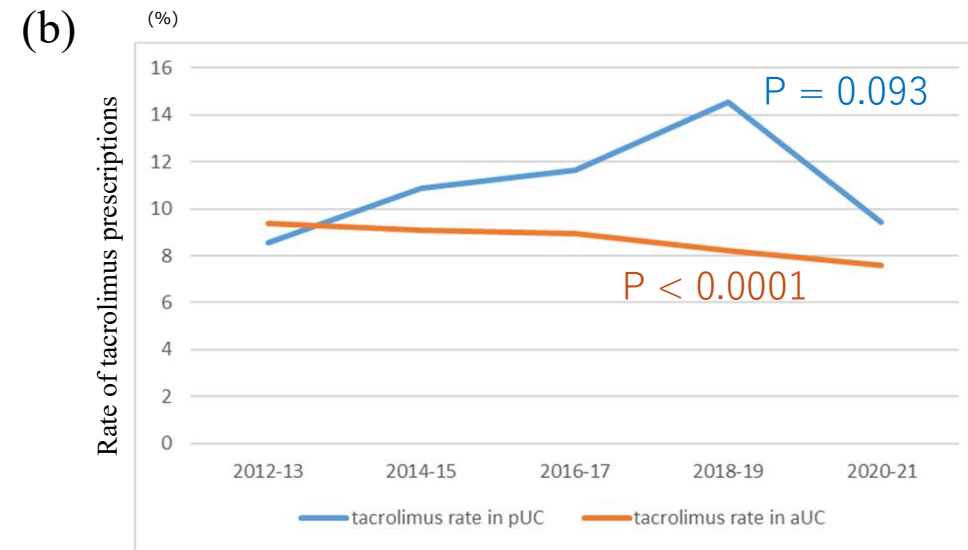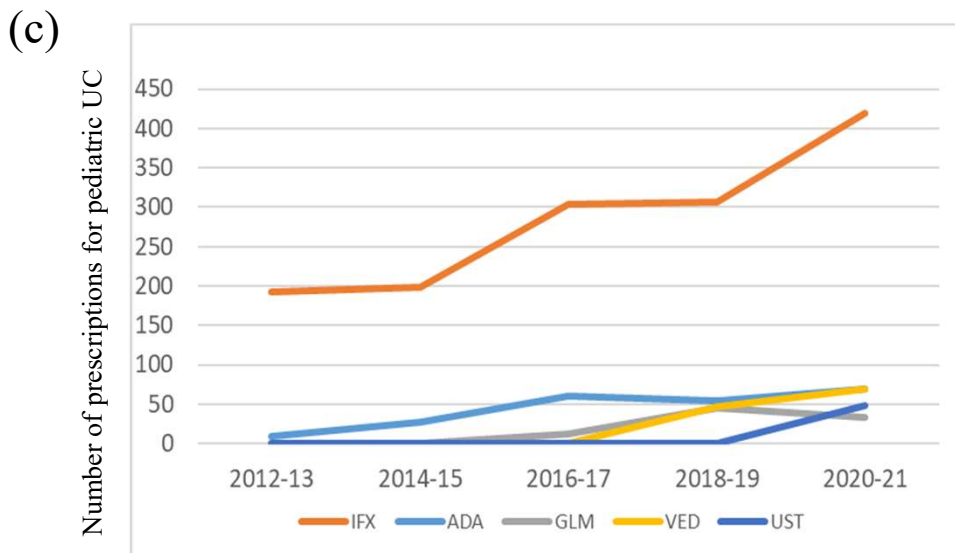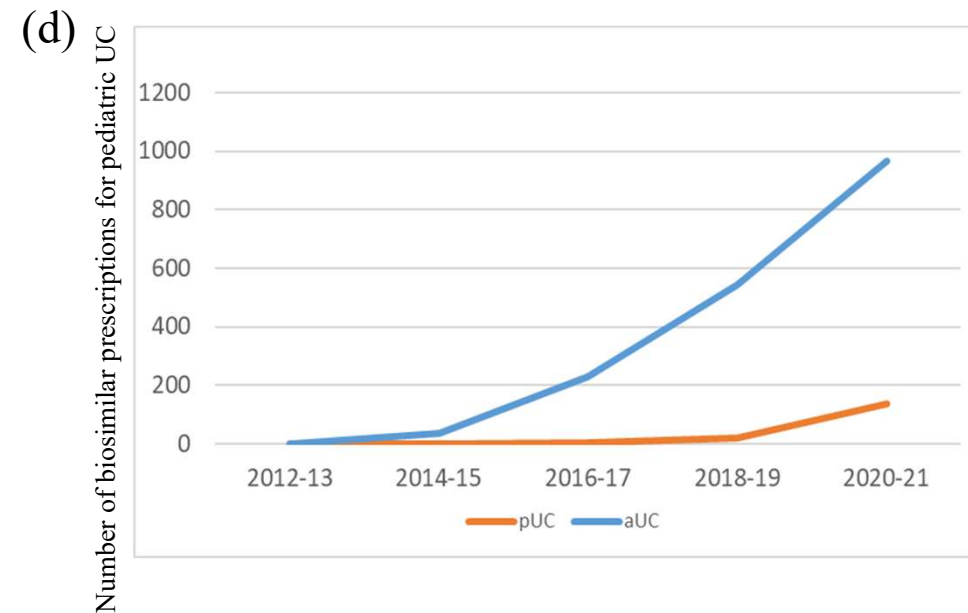

Supplementary figure 2

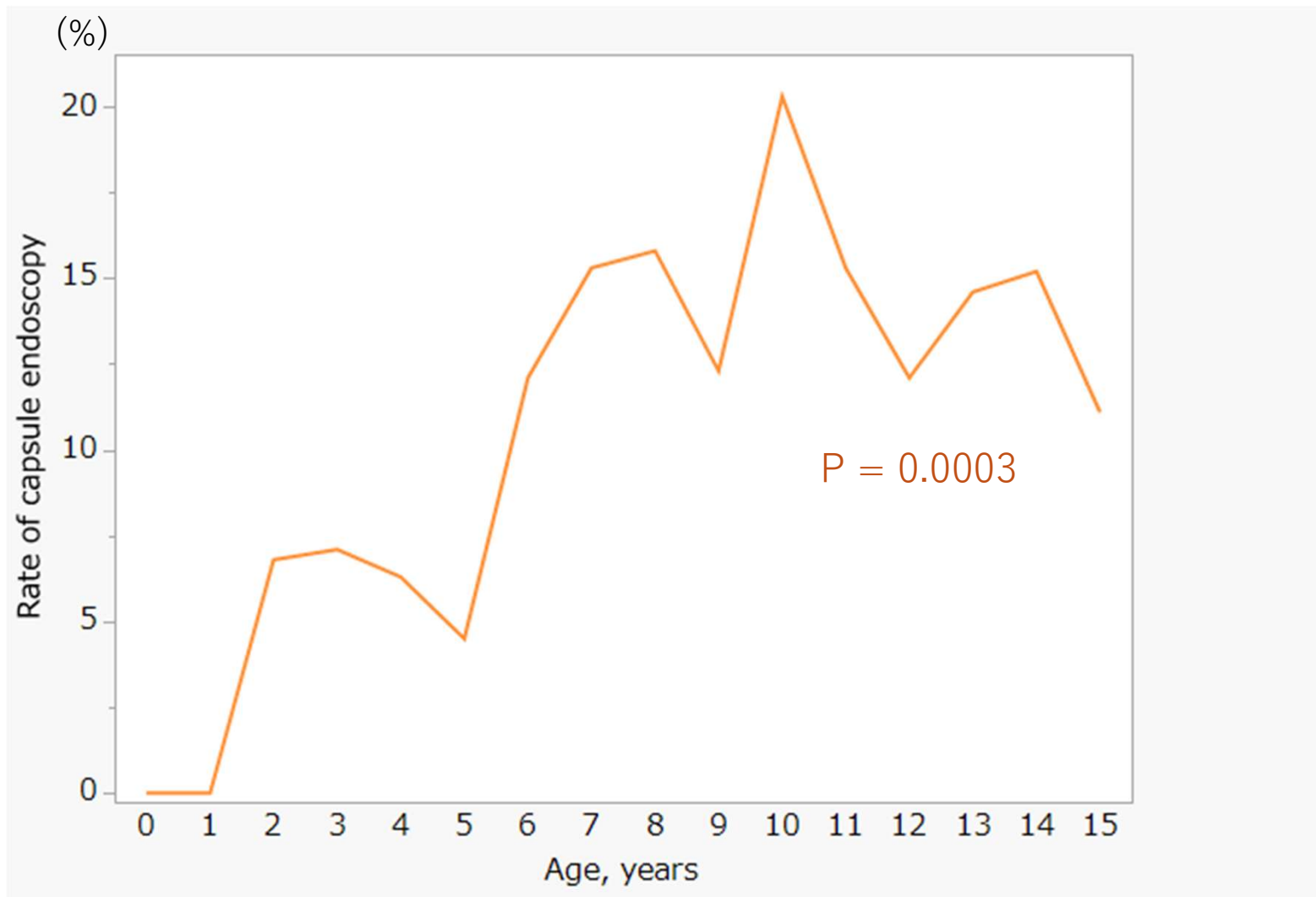

**Supplementary figure 3**

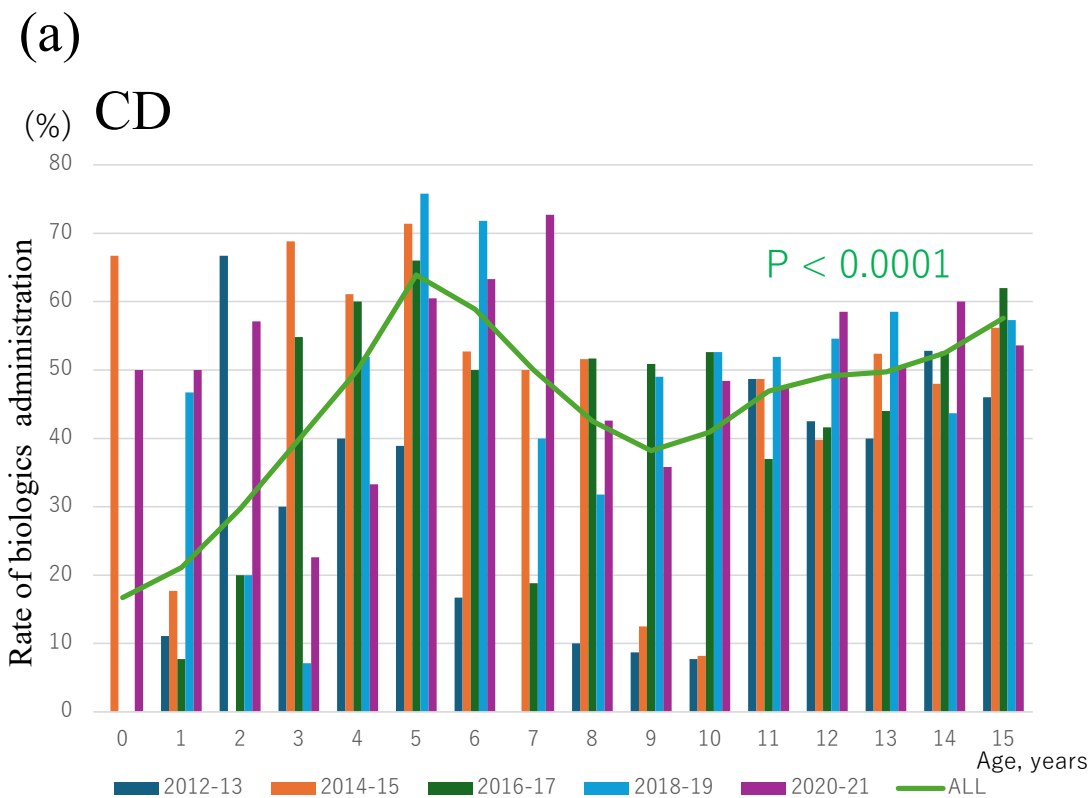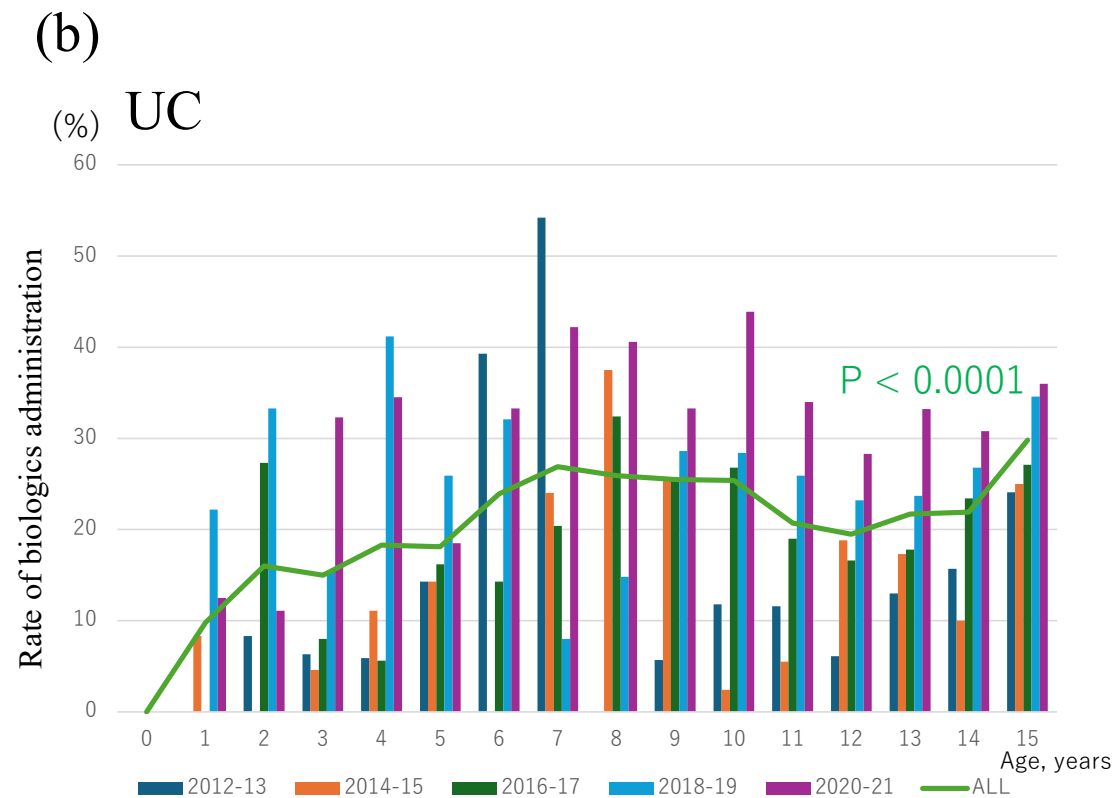

Supplementary figure 4

Supplement: Supplementary file 1 — Figure S1. Changes in Crohn’s disease management. (a) Number and rate of admissions of patients with CD as a proportion of admissions for all digestive diseases. The rate of admission of patients with CD for all digestive diseases significantly increased in both pediatric and adult patients (p < 0.0001 and p < 0.0001, respectively). (b) The rate of elemental diet prescriptions increased for pediatric and adult patients with CD (p < 0.0001, p < 0.0001, respectively). (c) The number of prescriptions for all biologics, particularly infliximab, had increased. (d) The number of biosimilar prescriptions for pediatric patients increased marginally. Figure S2. Changes in ulcerative colitis management. (a) The number of admissions for pediatric patients did not change. The admission rates of pediatric patients with UC as a proportion of admissions for all digestive diseases decreased (p < 0.0001). (b)The rate of tacrolimus prescriptions did not show an increasing tendency (p = 0.092). (c) The number of prescriptions for all biologics, particularly infliximab, has increased. (d) The number of biosimilar prescriptions for pediatric patients increased marginally. Figure S3. Correlation between patient age at admission and the use of capsule endoscopy. The Cochran–Armitage trend test demonstrated that the rate of capsule endoscopy usage increased significantly with advancing patient age (p = 0.0003). Figure S4. Trend of biologic administration in pediatric patients with IBD. (a) Patients with CD aged > 10 years exhibited a relatively stable and high rate of biologic administration over time. In contrast, the administration rates of biologics in patients aged ≤ 10 years showed a gradual increase over time. Furthermore, the Cochrane–Armitage trend test indicated a significant positive trend, with the rate of biologic administration increasing as patient age advanced (p < 0.0001). (b) Among pediatric patients with UC, the rate of biologic administration demonstrated an increasing t [file JGH3-9-e70175-s001.pdf]
